# SpaTemHTP: A Data Analysis Pipeline for Efficient Processing and Utilization of Temporal High-Throughput Phenotyping Data

**DOI:** 10.3389/fpls.2020.552509

**Published:** 2020-11-20

**Authors:** Soumyashree Kar, Vincent Garin, Jana Kholová, Vincent Vadez, Surya S. Durbha, Ryokei Tanaka, Hiroyoshi Iwata, Milan O. Urban, J. Adinarayana

**Affiliations:** ^1^Centre of Studies in Resources Engineering, Indian Institute of Technology Bombay, Mumbai, India; ^2^Crop Physiology, International Crop Research Institute for Semi-Arid Tropics (ICRISAT), Hyderabad, India; ^3^Institut de Recherche pour le Développement (IRD) – Université de Montpellier – UMR DIADE, Montpellier, France; ^4^Laboratory of Biometrics and Bioinformatics, University of Tokyo, Tokyo, Japan; ^5^Bean Physiology - Agrobiodiversity, Alliance of Bioversity International and CIAT, Cali, Colombia

**Keywords:** high-throughput phenotyping, SpATS, cross-validation, simulation, change point analysis, HTP-pipeline

## Abstract

The rapid development of phenotyping technologies over the last years gave the opportunity to study plant development over time. The treatment of the massive amount of data collected by high-throughput phenotyping (HTP) platforms is however an important challenge for the plant science community. An important issue is to accurately estimate, over time, the genotypic component of plant phenotype. In outdoor and field-based HTP platforms, phenotype measurements can be substantially affected by data-generation inaccuracies or failures, leading to erroneous or missing data. To solve that problem, we developed an analytical pipeline composed of three modules: detection of outliers, imputation of missing values, and mixed-model genotype adjusted means computation with spatial adjustment. The pipeline was tested on three different traits (3D leaf area, projected leaf area, and plant height), in two crops (chickpea, sorghum), measured during two seasons. Using real-data analyses and simulations, we showed that the sequential application of the three pipeline steps was particularly useful to estimate smooth genotype growth curves from raw data containing a large amount of noise, a situation that is potentially frequent in data generated on outdoor HTP platforms. The procedure we propose can handle up to 50% of missing values. It is also robust to data contamination rates between 20 and 30% of the data. The pipeline was further extended to model the genotype time series data. A change-point analysis allowed the determination of growth phases and the optimal timing where genotypic differences were the largest. The estimated genotypic values were used to cluster the genotypes during the optimal growth phase. Through a two-way analysis of variance (ANOVA), clusters were found to be consistently defined throughout the growth duration. Therefore, we could show, on a wide range of scenarios, that the pipeline facilitated efficient extraction of useful information from outdoor HTP platform data. High-quality plant growth time series data is also provided to support breeding decisions. The R code of the pipeline is available at https://github.com/ICRISAT-GEMS/SpaTemHTP.

## Introduction

During the last decade, progress in phenotyping methods have given ground for the development of many high-throughput phenotyping (HTP) platforms (Berger et al., 2010; Tisne et al., 2013; Cabrera Bosquet et al., 2015; Vadez et al., 2015), established across the globe to support rapid screening of plant phenotypes. These platforms generate large-scale phenotypic datasets that are complex to handle, process, and interpret. An important aspect that contributes to the complexity of HTP data handling is the presence of exogenous effects, which primarily include system-generated noise and fluctuations in environmental conditions. This is particularly the case in HTP platforms characterizing phenotypes in open-environments or under non-controlled conditions like the LeasyScan, the PhenoField (Barker et al., 2016), or the Field scanalyzer (Virlet et al., 2017) platforms. These effects can result in large heterogeneity besides erroneous and missing observations. Hence, the data treatment from outdoor platforms requires a systematic approach to deal with such artifacts and facilitate routine usage of HTP data (Dupuy et al., 2017; Namin et al., 2018; Tello et al., 2018). There are few existing automated procedures (pipelines) to process and analyze HTP data (Klukas et al., 2014). In addition, existing ones largely use image-based analysis of plant phenotypes (Hartmann et al., 2011; Artzet et al., 2019), and some are even platform or trait-specific (Galkovskyi et al., 2012; Faroq et al., 2013; Hasan et al., 2018).

This paper presents an automated data analysis pipeline called SpaTemHTP that processes and analyzes large temporal HTP data taking into consideration the specificity of data generated on outdoor platforms. The general philosophy of our pipeline is to progressively increase the information content of the data by applying a succession of analytical steps, which subsequently enhances the understanding of complex biological processes (Van Eeuwijk et al., 2019). The pipeline comprises three components: (a) data preprocessing; (b) genotype adjusted mean computation with spatial adjustment; and (c) further analysis of the genotype adjusted means time series by logistic curve fitting and change-point analysis.

Data generated in outdoors or field-based HTP platforms like the LeasyScan (Vadez et al., 2015) are subjected to conditions that result in occasional inaccuracies/failure of data-generation resulting in extreme or missing values. Therefore, the first step of our pipeline was to preprocess raw data by removing outliers and imputing missing values. We hypothesized that those steps increase the quality of the genotype adjusted mean computation, which is the crucial task of the pipeline. The detection of outliers prevents the model estimates from being impacted by extreme values that are inflated or wrong. Removing outliers also positively influences the imputation step by restricting the data distribution to a more realistic observation from which candidate values will be chosen. In a similar way, the imputation of missing values can help the estimation of the mixed model estimates by providing complete data that were imputed taking the temporal dimension into consideration. The use of imputation methods in longitudinal data has been shown to have a positive effect on the accuracy of the mixed-model estimates (Huque et al., 2018).

The second and main step of the pipeline was to calculate genotype adjusted means using spatial adjustment. The need to adjust phenotypic data for heterogeneity due to field variation is known since many years (Gilmour et al., 1997). So far, spatial adjustment was generally done by a sequential procedure of model fitting and diagnostics steps, which was difficult to implement in an automated way. More recently, Rodríguez-Álvarez et al. (2016) introduced the SpATS model, a two-dimensional (2D) P-spline approach for mixed model spatial adjustment. Velazco et al. (2017) demonstrated that the SpATS model could be successfully adapted for routine applications. Therefore, the SpATS model gave us the opportunity to perform spatial adjustment for genotype adjusted mean computation in an automated way on large HTP time series data. To the extent of our knowledge, the routine application of spatial adjustment to obtain genotype adjusted mean time series from outdoor platform HTP data has not yet been done.

Another advantage of the SpATS model application is the possibility to improve the estimation of the trait heritability, which is an important criterion for breeders (Velazco et al., 2017). In this article, we have considered the broad-sense trait heritability defined as *h*^2^ = $\sigma_{g}^{2}$$/(\sigma_{g}^{2}$ + $\sigma_{e}^{2}$), where $\sigma_{g}^{2}$ and $\sigma_{e}^{2}$ represent genotypic and error variance, respectively. Therefore, accounting for spatial variation will reduce $\sigma_{e}^{2}$ since some variation that was previously considered random noise will be considered spatial variation. Such a reduction of $\sigma_{e}^{2}$ would increase *h*^2^ and help obtain a better estimate of the proportion of the genetic component that resulted in phenotypic variability.

In the last step of the pipeline, we continued the temporal analysis of the genotype adjusted means generated in the previous step by modeling the growth curve and by identifying important plant growth stages. Indeed, temporal analysis of HTP data has also been rarely considered, although it is known that phenotypic expressions of genotypes vary with crop growth stages (Coleman et al., 1994; Ibañez et al., 2017). Plant growth typically follows temporal patterns that can be generally described as (i) initial slow growth (lag phase), (ii) phase of rapid (exponential) growth which gradually slows down toward the end of the system cycle, and (iii) steady phase (Yin et al., 2003; Shi et al., 2016). Phenotypic variations across these stages (i.e., the growth patterns) also contribute to the differences among the genotypes and influence genotypic adaptation to environmental contexts (Chapman, 2008; Kholová et al., 2014). These growth phases can be statistically detected and used to analyze genotypic variance (Chapman, 2008). Here, we used a change point analysis to identify the portion in the temporal dataset where the probability to capture genotypic variance was maximized. Therefore, the second important contribution of this study was the use of genotype adjusted means time series and heritability estimates (obtained after spatial correction) to enable the systematic identification of critical growth phase(s) having maximum genotypic variance.

In the following sections, SpaTemHTP is illustrated by applying it on a wide range of scenarios varying in terms of species (chickpea, sorghum), phenotypic traits (leaf area, projected leaf area, plant height), and experiment replications. Using real-data analyses and simulations, we evaluated the different components of SpaTemHTP (outlier detection, missing value imputation, and spatial adjustment) to estimate their relative contribution in the quality of the temporal series of genotypic estimates. We further illustrated the capacity of SpaTemHTP to detect growth phase and cluster genotypes in consistent groups.

## Materials and Methods

### Data and Test-Site Description

Phenotypic data from two diversity panels were used (Table 1). The first was a chickpea (legume) panel comprising 288 genotypes, and the second was a sorghum (cereal) panel with 384 genotypes. Those panels cover around 90% of the genetic diversity of their respective species (Upadhyaya et al., 2002; Billot et al., 2013). The two populations were phenotyped at ICRISAT-Patancheru (17.5111° N, 78.2752° E) using the LeasyScan HTP platform (Vadez et al., 2015)^1^ during two seasons. Information about the season, date, and the duration of each experiment is given in Table 1. In each experiment, four replicates of each genotype entry were laid out in an alpha design with 12 blocks containing 24 and 32 genotypes for the chickpea and the sorghum population, respectively. The crops were raised in plots of dimensions: 60 × 40 × 65 cm^3^ (length, width, and height) filled with farm-collected vertosol using agronomic practices recommended by Trivedi (2008). Here, we want to emphasize that using four replicates per genotype is larger than what is generally done in phenotyping experiments of similar association panels, e.g., in Rodríguez-Álvarez et al. (2016), two replicates were used, and in Zaman-Allah et al. (2015) and Prom et al. (2019), three replicates were used.

**TABLE 1:** Dataset description.

**Experiment**	**Date**	**MinT (°C)**	**MaxT (°C)**	**RH (%)**	**Min (%)**	**Max (%)**	**Mean (%)**
Chickpea E1 (CPE1)	25 Nov 2014–17 Dec 2014	13.15	29.02	89.73	0.000	52.080	5.797
Chickpea E2 (CPE2)	01 Dec 2015–07 Jan 2016	13.97	31.22	89.78	1.040	56.250	17.434
Sorghum E1 (SGE1)	15 Mar 2015–06 Apr 2015	18.42	40.67	45.84	0.000	2.210	0.293
Sorghum E2 (SGE2)	22 Oct 2015–12 Nov 2015	14.02	30.51	89.67	0.000	26.560	1.456

*The duration of data collection, the minimum and maximum recorded temperatures (MinT, MaxT) in degree Celsius (°C), and the average relative humidity (RH%) during the experiments (E) are shown for each crop (chickpea, sorghum) and experiment data, along with the minimum (Min), maximum (Max), and mean percentage of daily amount of missing data points.*

In the LeasyScan HTP platform (see schematic visualization in Supplementary Figure 1), Phenospex’s 3D laser scanners are employed to provide 3D images of the plants. The platform has a total capacity of around 5,000 sectors arranged in eight trenches (each having two columns). There are eight scanners (one per trench) mounted on top of an irrigation boom (automated sprinkle irrigation is used for the platform) which project a laser line on top of the canopy. A camera with 45° angle of view captures the reflection of the laser line at a high rate (50–80 pics/s), allowing the simultaneous reconstruction of 3D images of all the plants. Several algorithms then operate (handled by Phenospex engineers) to extract several morphological traits. The scanners measure the canopy parameters of each sector at an interval of 2 h, and the median value per day is used as the daily measurement of a given trait. Among the available traits, we used the daily means of 3D-reconstructed leaf area (LA3D; mm^2^), projected leaf area of the canopy (PLA; mm^2^), and plant height (PH; cm). Those traits indicate the rates of biomass accumulation and can be considered an estimate of crop vigor (Flood et al., 2016; Sivasakthi et al., 2017; Yang et al., 2020).

### Pipeline Overview

**FIGURE 1:**
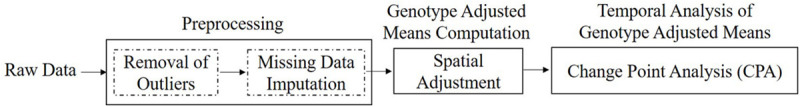
Block diagram of the three stages of SpaTemHTP pipeline, illustrated according to the sequence of steps followed for HTP data analysis.

The pipeline outline is illustrated in Figure 1 which sequentially represents the discrete modules used for converting raw phenotypic data into useful information. The following sections describe each of the methods used for data preprocessing, genotype adjusted mean computation, and temporal analysis of genotypic estimates.

### Stage 1: Preprocessing

#### Outlier Detection

The first step in preprocessing was the detection of outliers, which are the extreme values that occurred mostly due to measurement errors. Outliers in the raw data were detected for each day using boxplots (Sun and Genton, 2011) of the phenotypic value distribution per day, i.e., the distribution included all genotype and replicate values of a specific day. The 25% quantile (QR1), 75% quantile (QR3), and 50% interquantile range (IQR) were calculated, and the observations below QR1 – 1.5 ^∗^ IQR or above QR3 + 1.5 ^∗^ IQR were considered outliers. Those outliers were replaced by missing values and then imputed in the next step along with the already existing missing values in the dataset.

#### Missing Value Imputation

The second step in preprocessing was the imputation of missing values. According to Huque et al. (2018), the use of multiple imputations (MI) on longitudinal data can improve the accuracy of mixed model estimates. This can be due to the capacity of MI to borrow information on the past and future points of the time series to identify genotypes with similar growth pattern and fill the gap of the missing genotypes with similar observed values. We performed MI using the predictive mean matching (PMM) method from the R package “mice” (Buuren and Groothuis-Oudshoorn, 2010). PMM is a method that was developed to reduce the bias by drawing real values sampled from the observed data (Rubin and Schenker, 1986). Therefore, the detection of outliers prior to imputation helps in restricting the observed values distribution to credible values by removing extreme observations. PMM is also a robust and an assumption free method, which can be used for traits with any type of distributions (White et al., 2011).

In our situation, PMM sequentially imputes the missing values of a specific day using the other days information by applying the following steps:

(a) Let us assume we impute the missing values of *d*_*i*_. In that case, the other days constitute the set of predictors, *z* = [*d*_1_, …, *d*_*(i–1)*_, *d*_*(i+1)*_, …, *d*_*n*_].
(b) For the non-missing values of *d*_*i*_, PMM performs a linear regression of *d*_*i*_ on *z*: *d*_*i*_ = *b*${}_{1}^{*}$d_1_ + … + *b*${}_{\left(i-1\right)}^{*}\,d_{\left(i-1\right)}$ + *b*${}_{\left(i+1\right)}^{*}\,d_{\left(i+1\right)}$ + … + *b*_*n*_^∗^*d*_*n*_ using the complete time series information to determine phenotypic values with similar biological trends.
(c) PMM samples a reduced number of linear regression coefficients from the whole sample **b** = [*b*_1_, *b*_2_, …, *b*_*n*_] to predict values for both missing ${\hat{y}}_{{\mathrm{di}}_{M\,i\,s\,s}}$ and non-missing values of *d*_*i*_ ${\hat{y}}_{{\mathrm{di}}_{O\,b\,s}}$. The random sampling of the **b** coefficients allows to generate some variability by sampling in the multivariate normal distribution with mean **b** variance σ^2^ (**b**).
(d) For each predicted value corresponding to a missing value ${\hat{y}}_{{\mathrm{di}}_{M\,i\,s\,s}}$, PMM identifies a number of close values in ${\hat{y}}_{\mathrm{di}}$ (in our case 5) corresponding to an observed value *y*_di_Obs__. Those values can come from any observed data point of the ${\hat{y}}_{{\mathrm{di}}_{O\,b\,s}}$ distribution.
(e) PMM imputes each missing values *y_di_Miss__* by drawing one of the observed values *y_diO_bs__* from the sample of values for which the predictions ${\hat{y}}_{{\mathrm{di}}_{O\,b\,s}}$ were close enough to the missing value prediction ${\hat{y}}_{{\mathrm{di}}_{M\,i\,s\,s}}$.
(f) The procedure is repeated for each missing value of *d*_*i*_ and for each day. For further details, see van Buuren and Groothuis-Oudshoorn (2009).

PMM works with data missing at random (MAR) or missing completely at random (MCAR) (Morris et al., 2014). In our case, the daily amount of missing values ranged between 0 and 56.25% with an average of 6.25% across the eight configurations (crop × experiment × traits) (Table 1). We noticed that missing data did not show any pattern across time (see Supplementary Figure 2). Therefore, we could reasonably assume that data were not missing not at random (MNAR) because the missing pattern did not depend on the plant or trait growth stage. In the case of the LeasyScan, which is set up outdoors with partly wireless data transmission, we assumed that data were mostly missing at random being a mix of: (a) MCAR data due to external factors like technical problems and/or natural phenomenon (e.g., wind, birds, or other animals) and (b) MAR data due to time and spatial position. In the latter case, temporal and spatial information was taken into consideration in the PMM imputation process. According to Marshall et al. (2010), the PMM algorithm can handle up to 75% of missing values.

We hypothesize that the sequential application of outlier detection and PMM missing value imputation in HTP time series data is a simple strategy to replace extreme values by realistic observations using the information from the growth pattern, which will ultimately improve the accuracy of the genotype mixed model estimates.

### Stage 2: G-BLUE Computation With Spatial Adjustment

The second step of the pipeline was the computation of genotype best linear unbiased estimates (G-BLUEs) using spatial adjustment. The G-BLUEs were calculated using the following realization of the SpATS model (Rodríguez-Álvarez et al., 2016; Velazco et al., 2017):

$$y_{\mathrm{ijklm}}=\mu +r\,e\,p_{j}+b\,l\,o\,c\,k_{k\,\left(j\right)}+f\,\left(r\,o\,w_{l},c\,o\,l_{m}\right)$$

(1) $$\;+r\,o\,w_{l}+c\,o\,l_{m}+g\,e\,n\,o_{i}+e_{\mathrm{ijklm}}$$

where, *y_ijklm_* is the trait value of *j*th replication of genotype *i* in block *k*, row *l*, and column *m*. The term *f*(*row*_*l*_,*col*_*m*_) is an expression of the smooth spatial surface expressed in terms of row and column information accounting for spatial variation. In the SpATS model, *f*(*row*_*l*_,*col*_*m*_) is modeled by a 2D-penalized spline including linear trends across rows and columns, row-column linear interaction, smooth trend across rows and columns, and a smooth-by-smooth interaction between rows and columns. For further details, see Rodríguez-Álvarez et al. (2016) and Velazco et al. (2017). Finally, *e_ijklm_* represents the plot error term that is normally distributed $N\,\left(0,\sigma_{e}^{2}\right)$. The replicate, block, row, and column terms were considered random with a specific error term. To calculate the G-BLUEs, the genotype term was treated as fixed.

To evaluate the effect of spatial adjustment, we calculated the G-BLUEs with and without spatial adjustment. The G-BLUEs without spatial adjustment were calculated using a reduced version of model 1 without the *f*(*row*_*l*_,*col*_*m*_) term. In a similar way, we used those two versions of model 1 (with and without spatial term) to calculate the heritability (*h*^2^). In those cases, we treated the genotype term as random to estimate the genetic variance $\sigma_{g}^{2}$. For the non-spatially adjusted model, *h*^2^ = $\sigma_{g}^{2}$$/(\sigma_{g}^{2}+$$\sigma_{e}^{2}$). For the spatially adjusted model, *h*^2^ is calculated in terms of the effective dimension or the effective degrees of freedom associated to the genetic component in the SpATS model (ED_*g*_) (Rodríguez-Álvarez et al., 2016). The effective dimension of a model is computed as the trace of the hat matrix *H*, and Hg is the hat matrix for genotypes. If the number of genotypes = *n*_*g*_ and the number of zero eigenvalues of *H*_*g*_ = l, then *h*^2^ = ED_*g*_/(*n*_*g*_−*l*). We calculated models with spatial adjustment using the R package “SpATS” (Rodríguez-Álvarez et al., 2016) and the one without spatial adjustment in GenSTAT version 18 (VSN International, 2015).

The combination of each of the abovementioned data processing and G-BLUE calculation options: outlier detection (yes/no), missing value imputation (yes/no), and spatial adjustment (yes/no), represents a total of eight strategies (S1–S8) to calculate G-BLUEs from raw data (Table 2). In the next part of the article, we will refer to those strategies as S1–S8.

**TABLE 2:** List of strategies to process data from the raw data to the G-BLUE computation.

**Strategy**	**Outlier detection**	**Missing value imputation**	**Spatial adjustment**
S1	✗	✗	✗
S2	✓	✗	✗
S3	✗	✓	✗
S4	✓	✓	✗
S5	✗	✗	✓
S6	✓	✗	✓
S7	✗	✓	✓
S8	✓	✓	✓
S9	Single-step mixed model		

*S1–S8 represent a combination of use (✓) or no use (✗) of outlier detection, missing value imputation, and spatial adjustment. S9 is an iterative strategy for spatial adjustment where missing values are imputed during the mixed model computation and outliers detected using the mixed model residuals.*

### Single-Step Mixed-Model G-BLUE Computation

An alternative to generate the G-BLUEs is to use a single-step mixed-model approach where outliers are iteratively removed based on the model residuals and the missing values imputed during the estimation procedure. We called this strategy S9 and compared it with the other strategies (S1–S8). To perform S9, outliers were iteratively removed by applying the Grubbs test (*p* < 0.05) (Grubbs, 1950) on the residuals of model 1. This strategy is similar to the one applied in Lehermeier et al. (2014).

### Validation of the G-BLUE Computation

To validate our pipeline, the G-BLUE computation and the data preprocessing steps were extensively evaluated. We performed cross validation (CV) to assess the predictive ability of the mixed model used for the G-BLUE and heritability (*h*^2^) computation, which are important information for breeding purposes. We also performed a more direct evaluation of the G-BLUE computation by estimating the correlation between the G-BLUEs obtained from the same population but in two different experiments (e.g., CPE1 and CPE2) at the same stage of development.

#### Cross Validation

For each combination of population (chickpea, sorghum), trait (PH, LA3D), and experiment (E1, E2), 10 replications of a fivefold CV were performed. To cover the time variability, a different day for each CV replication was randomly selected. In each CV replication, the trait observations were randomly assigned to five samples of equal size. During the five runs, each sample was successively used as the validation set, and the rest of the data went into the estimation set. The estimation set was used to estimate the parameters of model 1. Then, using those parameters estimates, validation set trait values were predicted (${\hat{Y}}_{\mathrm{VS}}$) as per the experimental design and genotype information of the validation set. We used the Pearson correlation (ρ_$\hat{y}$, *y*_) between (${\hat{Y}}_{\mathrm{VS}}$) and the observed trait values as a measure of the predictive ability. During the CV, the *h*^2^ in the training sets was also estimated to evaluate the influence of the procedure options on this parameter.

For each CV run, we sequentially applied S1–S9 preprocessing and spatial adjustment strategies on the same estimation and validation data partitions. This allowed us to evaluate the contribution of each component (outlier detection, missing value imputation, and spatial adjustment) on the predictive ability of the mixed model used to calculate the G-BLUEs and the heritability. Using 10 replications of a fivefold CV gave 50 values to determine the average ρ_$\hat{y}$, *y*_ and *h*^2^ of each strategy.

#### Between Experiment Comparison

To complement the CV, we also performed a comparison between experiments run on the same population but at different times, for example, CPE1 and CPE2. For each day at a comparable growth stage, we calculated the Pearson correlation (ρ_*E1, E2*_) between the G-BLUEs in the two experiments. ρ_*E1, E2*_ evaluates the ability of a strategy to estimate accurately the genotypic component of the phenotype measurements (Velazco et al., 2017). Ideally, we expect this genetic component to be stable across experiments.

The average ρ_*E1, E2*_ was calculated for each combination of trait (PH, LA3D) and pair of experiments (CPE1–CPE2, SGE1–SGE2) over all comparable days (CP, 22 days; SG, 21 days). This operation was repeated for all Table 2 strategies to evaluate the respective influence of outlier detection, missing data imputation, and spatial adjustment on ρ_*E1, E2*_.

#### Simulations

As suggested by one of the reviewers, we performed simulations based on real data to evaluate the robustness of the methods to manage outliers and/or missing values in the G-BLUE computation. For that purpose, the SGE1 PH data was selected as the most “controlled” dataset, since it had the lowest percentage of missing values (0.29%), a low percentage of outliers (1.6%), and a high heritability (around 0.7) showing the importance of the genetic component compared with other factors. The 15th day of the series was selected as the reference since it contained no missing values and a low number of outliers (1.4%). The G-BLUEs of that day were calculated and kept as reference. Then some noise was introduced in the time series by adding extreme and/or missing values. We tested three scenarios: (a) addition of *x* percent of missing values; (b) addition of noise on *x* percent of the values to generate outliers; and (c) a + b. To generate outliers, we added values drawn from a normal distribution *N*(0, 3^∗^σ^2^(population at day *i*)) to *x* percent of the data. The simulations were run setting *x* at 10, 20, 30, 40, and 50%. We evaluated the efficiency of the outlier detection, missing value imputation, and their combination by calculating the correlation between the reference G-BLUEs and the G-BLUEs obtained with strategies S5–S9. We repeated the procedure 10 times for each scenario.

#### Assessment of the Genotype Growth Patterns

Time-ordered plots of G-BLUEs obtained with strategies S5–S9 were used to visualize the general growth pattern of the genotypes. We also performed some modeling of the growth curves by fitting a logistic curve to the genotype-specific G-BLUE time series. The logistic curve can be used to describe data with a sigmoidal pattern as the one we expect in biological growth (Cao et al., 2019). To fit the logistic curve, the function “drm” from the “drc” R package (Ritz et al., 2015) was used, which determines the curve parameters by likelihood function maximization. The average coefficient of determination (*R*^2^) was used as a goodness-of-fit measurement to evaluate the possibility to summarize the G-BLUEs data with the logistic function for each combination of species, trait, experiment, and strategy (S5–S9).

### Stage 3: Temporal Analysis of G-BLUEs

In the LeasyScan platform, it has been observed that the scanner resolution drops as plants grow larger and canopies overlap with each other (Vadez et al., 2015). At this stage, while trait value differences between genotypes increase, the *h*^2^ tends to decrease (Vadez et al., 2015). Both trait differences and *h*^2^ represent two key components for breeders to make selection. They need to select from maximum trait differences (canopy growth traits here), while having maximum genetic variance (highest possible *h*^2^). It was thus, important to identify the time-points (or duration) at which the progression and relationship between both the variables altered together. Additionally, we also wanted to identify a window, called the optimal time window (OTW) in the crop growth duration which maximizes genotypic diversity as well as *h*^2^. To obtain the OTW, simultaneous changes in the distributions of the two variables during crop growth were first identified using the multivariate change-point analysis (CPA) method (Matteson and James, 2014). CPA was performed for each trait separately.

To represent the evolution of the genetic diversity over time, we used a measurement of distance between genotype cluster (Clust-Dist). Clust-Dist was calculated through clustering of the entire spatially adjusted G-BLUEs time series (of each trait) using the Gaussian Kernel K-Means clustering method (Dhillon et al., 2004; Steinbach et al., 2004). To determine the optimal number of genotypic clusters, the Silhouette method (Rousseeuw, 1987; Das and Padhy, 2017) was used. After determining the optimal number of clusters, genetic diversity values between those clusters were estimated by calculating the Euclidean distance between the cluster centers at each day (Dhillon et al., 2004). The Clust-Dist were leveraged as a measurement of genetic diversity change over time. The incorporation of distance between clusters as a measure of genotypic diversity has also been shown in Tyagi et al. (2015) and Sarker et al. (2017). Subsequently, the trait *h*^2^ calculated for each day resulted in a time-ordered set, that was used as the second variable for performing CPA. The E-statistic-based multivariate CPA was implemented to identify the TWs using the R package “ecp” (Matteson and James, 2014). The utilization of multivariate time series data for detecting and understanding the temporally changing relationships between different variables is also shown in Cabrieto et al. (2017).

### Genotypic Clusters × Time Window Analysis

A genotypic clusters (*G*_*c*_) × time window (TW) analysis was conducted as a validation of the final pipeline results. This analysis was used for understanding the relative importance of identified groups of genotypes, of the TWs, and of their interaction. For that purpose, we clustered the G-BLUEs within the OTW into three groups (“low,” “medium,” and “high”) using a k-means clustering procedure (Ding and He, 2004). A two-way analysis of variance (ANOVA) was performed to examine the stability of the clusters in terms of the statistical significance of the *G*_*c*_ × TW interaction for each trait. In this step, the average values of the G-BLUEs within each TW of each genotype, were used to estimate the interaction effects using the model: *y*_*ijk*_ = μ + *Gc*_*i*_ + *TW*_*j*_ + *Gc*×*TW*_*ij*_ + *e*_*ijk*_, where *y*_*ijk*_ is the average G-BLUE value of the genotypes present in *i*th genotypic cluster, *j*th time window and *k*th replicate. *Gc*_*i*_ and *T*_*j*_ are the effects of *i*th cluster and the *j*th TW, respectively. *G*_c_*Gc*×TW_*ij*_ denotes the interaction effect between the *i*th cluster and the *j*th TW. *e*_*ijk*_ represents the residual error term.

### Pipeline Code and Data Availability

The pipeline was programmed in an R (R Core Team, 2017) package available at: https://github.com/ICRISAT-GEMS/SpaTemHTP. All data, scripts, and functions required to reproduce the results can be found at: https://github.com/ICRISAT-GEMS/SpaTemHTP_Validation.

## Results

### Validation

#### Cross Validation

In Table 3, we present the average ρ_$\hat{y}$, *y*_ and *h*^2^ results obtained over the whole CV procedure. The information is organized per data treatment (outlier detection, missing value imputation, and spatial adjustment) such that each cell represents the average treatment effect, e.g., outlier detection yes over the two other treatments (i.e., missing value imputation: yes and no, and spatial adjustment: yes and no). For example, the first cell represents the average ρ_$\hat{y}$, *y*_ results of S2, S4, S6, and S8 (all strategies with outlier detection). For each treatment, the average ρ_$\hat{y}$, *y*_ difference with and without the treatment was calculated to evaluate its usefulness. The statistical significance of those differences was estimated using a *t*-test. At the end of Table 3, the difference between S8, which includes all data treatment options, and the single-step mixed-model G-BLUE computation (S9) is also compared.

**TABLE 3:** Average predictive ability (ρ_$\hat{y}$, *y*_) and heritability (*h*^2^) for LA3D and PH traits of chickpea (CP) or sorghum (SG) experiments 1 and 2 (E1, E2) obtained with (denoted as “yes”) and without (denoted as “no”) the effect of each data treatment (outlier detection, missing value imputation, and spatial adjustment (ρ_$\hat{y}$, *y*_).

**Predictive ability (ρ_$\hat{y}$, *y*_)**		**CP**				**SG**			
		**LA3D**		**PH**		**LA3D**		**PH**	
		**E1**	**E2**	**E1**	**E2**	**E1**	**E2**	**E1**	**E2**
Outlier detection	No	0.75	0.58	0.89	0.67	0.67	0.60	0.75	0.56
	Yes	0.75	0.57	0.88	0.69	0.67	0.56	0.75	0.56
	Difference	0.00	0.01	0.00	−0.03	0.00	0.04	0.00	−0.01
Missing value imputation	No	0.75	0.57	0.88	0.68	0.67	0.56	0.75	0.55
	Yes	0.75	0.59	0.88	0.69	0.67	0.60	0.75	0.56
	Difference	0.00	0.02	0.00	0.01	0.00	0.04	0.00	0.01
Spatial adjustment	No	0.70	0.54	0.88	0.68	0.65	0.50	0.71	0.52
	Yes	0.80	0.60	0.89	0.68	0.69	0.66	0.79	0.60
	Difference	0.10***	0.06**	0.00	0.00	0.04	0.16***	0.08**	0.08**
S8		0.81	0.64	0.89	0.70	0.69	0.67	0.78	0.60
S9		0.81	0.63	0.88	0.70	0.70	0.67	0.80	0.61
Difference		0.00	0.01	0.00	0.00	0.00	0.00	−0.02	−0.01

**Heritability (*h*^2^)**		**CP**				**SG**			
		**LA3D**		**PH**		**LA3D**		**PH**	
		**E1**	**E2**	**E1**	**E2**	**E1**	**E2**	**E1**	**E2**

Outliers removal	No	0.69	0.37	0.87	0.64	0.65	0.34	0.70	0.48
	Yes	0.69	0.35	0.79	0.74	0.65	0.34	0.73	0.51
	Difference	−0.01	−0.01	−0.08***	0.10***	0.00	0.01	0.03	0.04**
Missing value imputation	No	0.68	0.35	0.78	0.65	0.65	0.34	0.72	0.49
	Yes	0.69	0.37	0.87	0.74	0.65	0.34	0.71	0.50
	Difference	0.01	0.03	0.09***	0.09**	0.00	0.00	−0.01	0.01
Spatial adjustment	No	0.54	0.26	0.72	0.56	0.54	0.20	0.59	0.34
	Yes	0.84	0.45	0.94	0.82	0.75	0.48	0.83	0.65
	Difference	0.31***	0.19***	0.23***	0.26***	0.21***	0.28***	0.24***	0.31***
S8		0.85	0.47	0.94	0.88	0.75	0.49	0.84	0.70
S9		0.85	0.46	0.95	0.87	0.76	0.47	0.86	0.68
Difference		0.00	0.01	−0.01	0.01	0.00	0.01	−0.03	0.02

“Difference” corresponds to the average difference between the ρ_$\hat{y}$, *y*_ and h^2^ results obtained in scenarios with and without the data treatment. The difference between S8 and S9 is also shown for both ρ_$\hat{y}$, *y*_or h^2^. **p < 0.01; ***p < 0.001.

Concerning ρ_$\hat{y}$, *y*_, spatial adjustment was the only treatment that enabled a significant improvement in the results. The effect of spatial adjustment was significant in five out of eight cases. The significance of spatial adjustment was also noticed in terms of *h*^2^. The average *h*^2^ difference between strategy with and without spatial adjustment was always large and highly significant. For further details about the implementation of spatial adjustment for different traits see Supplementary Figures 3, 4. The effect of missing value imputation on *h*^2^ was also beneficial in some cases (CPE1 PH, CPE2 PH). Finally, from the comparison between the S8 and S9, it can be emphasized that there were no significant differences either in terms of ρ_$\hat{y}$, *y*_ or *h*^2^ in the CV procedure.

#### Between Experiment Comparison

Table 4 consolidates the results from the between experiment comparison procedure, and it is similarly organized as Table 3. The information per data treatment is presented such that each cell represents the average G-BLUEs correlation (ρ_*E1,  E2*_) for a specific treatment (e.g., remove outliers) over the other treatments (i.e., missing value imputation: yes and no, and spatial adjustment: yes and no). For example, the first cell represents the average ρ_*E1,  E2*_ for S2, S4, S6, and S8 (all strategies with outlier detection). For each treatment, the difference between the average ρ_*E1,  E2*_ with and without the treatment was calculated to evaluate the usefulness of each treatment. The statistical significance of those differences was estimated using a *t*-test. The end of Table 4 shows the comparison between S8 and S9.

**TABLE 4:** G-BLUEs correlation between two experiments on the same population (ρ_*E1,  E2*_) of chickpea (CP) and sorghum (SG), for the traits LA3D and PH, obtained with (denoted as “yes”) and without (denoted as “no”) the effect of each data treatment (outlier detection, missing value imputation, and spatial adjustment.

**G-BLUEs between experiments correlation (ρ_E1, E2_)**		**CP**		**SG**	
		**LA3D**	**PH**	**LA3D**	**PH**
Outlier detection	No	0.71	0.85	0.55	0.68
	Yes	0.66	0.90	0.54	0.67
	Difference	−0.04	0.05*	−0.01	−0.01
Missing value imputation	No	0.66	0.88	0.54	0.67
	Yes	0.69	0.87	0.55	0.68
	Difference	0.03*	−0.01	0.01	0.01
Spatial adjustment	No	0.65	0.84	0.53	0.65
	Yes	0.69	0.90	0.56	0.69
	Difference	0.04*	0.06*	0.03	0.04*
S8		0.72	0.90	0.57	0.70
S9		0.71	0.87	0.56	0.69
Difference		0.01	0.03*	0.01	0.01

“Difference” represents the average difference in **ρ**_*E1, E2*_ between strategies including or not the data treatment, as well as between S8 and S9. *p < 0.05.

The between experiment comparison results confirmed what we observed in the CV procedure. The most influential factor was the spatial adjustment. In three situations out of four, we observed a significantly larger ρ_*E1,  E2*_ in strategies with spatial adjustment. We could also observe a small positive effect with the detection of outliers (CP PH) and imputation of missing values (CP LA3D). Concerning the comparison between S8 and S9, we could observe a small positive effect of using S8 in the CP PH case.

#### Simulations

Table 5 contains the simulation results. As expected, the correlation between the calculated G-BLUEs and the reference decreased from the scenario with only missing values to the scenario with missing values and noise. We could also notice that the correlation decreased when the amount of missing values and/or noise increased from 10 to 50%. We could observe that, generally, the results obtained in the scenarios with only missing values were high and stable with values between 0.91 and 1. The result obtained with method S7 using only imputation were particularly good (0.99–1).

**TABLE 5:** Results of the simulation using real data (SGE1 PH).

	**Strategy**	***x* = 10%**	***x* = 20%**	***x* = 30%**	***x* = 40%**	***x* = 50%**
Add *x* missing values	S5	0.99	0.98	0.96	0.94	0.92
	S6	0.98	0.97	0.95	0.93	0.91
	S7	1	1	1	1	0.99
	S8	0.98	0.98	0.98	0.98	0.98
	S9	0.99	0.96	0.96	0.94	0.92
Add *x* noise values	S5	0.87	0.8	0.73	0.67	0.64
	S6	0.95	0.89	0.8	0.72	0.64
	S7	0.87	0.8	0.73	0.67	0.64
	S8	0.96	0.9	0.81	0.73	0.65
	S9	0.93	0.8	0.74	0.68	0.66
Add *x* missing values + *x* noise values	S5	0.84	0.68	0.57	0.48	0.39
	S6	0.93	0.79	0.6	0.47	0.38
	S7	0.85	0.72	0.62	0.52	0.43
	S8	0.94	0.83	0.68	0.51	0.43
	S9	0.88	0.69	0.58	0.47	0.4

*Correlation coefficient between 15th-day G-BLUE reference values and values from the simulation for the three scenarios: (a) addition of x percent of missing values, (b) addition of noise x percent of the values to generate outliers, (c) a + b, for strategies S5–S9.*

In the simulations with noise addition and noise addition along with missing values, we could observe that strategy S8, which combines outlier detection and missing value imputation, produced the largest correlation in almost all situations. In all those cases but one (addition of 50% of noise), S8 outperformed S9. The differences between the best method (S8) and the others increased until 20% of noise and/or missing values and then decreased.

#### Assessment of the Genotype Growth Patterns

**FIGURE 2:**
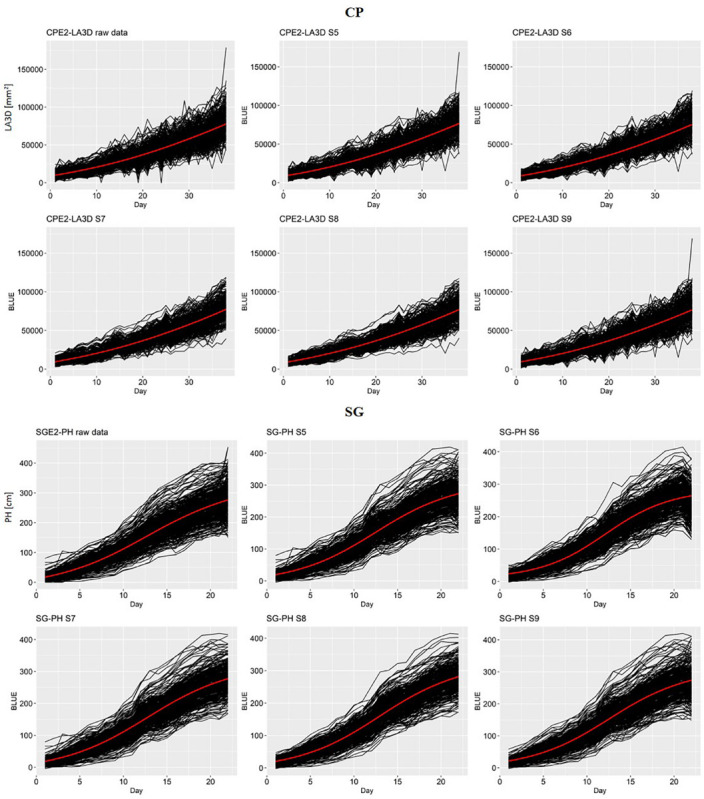
Comparison of the biological growth pattern of the raw data for chickpea (CPE2) LA3D and sorghum (SGE2) PH and the series of genotypic BLUEs obtained from S5 to S9. The red line in each plot represents the average of the fitted logistic curves for all genotypes.

In Figure 2, the raw genotype scores and the G-BLUEs time series obtained with different strategies (S5–S9) were plotted in an increasing order of data treatment: from raw data to S8 involving all data treatments. This figure helped to visually evaluate the quality of the time series in two specific scenarios (CPE2 LA3D and SGE2 PH). The G-BLUEs time series of the more erratic raw chickpea data was found to improve considerably from S5 to S8 with progressive introduction of the data treatment steps (outlier detection, imputation, and spatial adjustment). The data treatment sequence reduced the amount of abrupt fluctuations in the time series. In CPE2 LA3D, it was also observed that the G-BLUEs time series obtained from S8 were less erratic than the ones obtained with S9 (single-step mixed model).

An inspection of the SGE2 PH time series revealed that the phenotypic data were originally less noisy than the raw CPE2 LA3D values. The differences between the time series and the growth trend corresponding to the different scenario (S5–S9) were also less prominent than in CPE2 LA3D. From the general shape of SGE2 PH values and the average logistic function (red curve), it was evident that these data present a nice example of the sigmoidal growth pattern.

From a general point of view, we could notice in Table 6 that the G-BLUEs growth pattern was well described by the logistic curve. The growth patterns of the sorghum experiments (*R*^2^: 0.97–0.99) and chickpea LA3D (*R*^2^: 0.94–0.98) were almost perfectly described by the logistic curve. For chickpea PH, 71–85% of the variation could be explained by the logistic curve. In all situations, the use of strategy S8 combining outlier detection and missing value imputation produced the highest *R*^2^ values. In the chickpea PH experiments, we could see that strategy S6 using outlier detection performed better than S7 with only missing value imputation.

**TABLE 6:** *R*^2^ estimates of the logistic fit for the G-BLUEs time series data obtained with strategies S5–S9 for traits, LA3D, and PH of each chickpea (CP) and sorghum (SG) experiments.

**Strategy**	**CP**				**SG**			
	**LA3D**		**PH**		**LA3D**		**PH**	
	**E1**	**E2**	**E1**	**E2**	**E1**	**E2**	**E1**	**E2**
S5	0.94	0.95	0.71	0.8	0.98	0.97	0.99	0.99
S6	0.95	0.95	0.77	0.85	0.98	0.98	0.99	0.99
S7	0.95	0.98	0.73	0.81	0.98	0.98	0.98	0.99
S8	0.95	0.98	0.79	0.87	0.98	0.98	0.99	0.99
S9	0.94	0.95	0.74	0.83	0.98	0.97	0.98	0.99

### Change-Point Analysis of G-BLUEs

**FIGURE 3:**
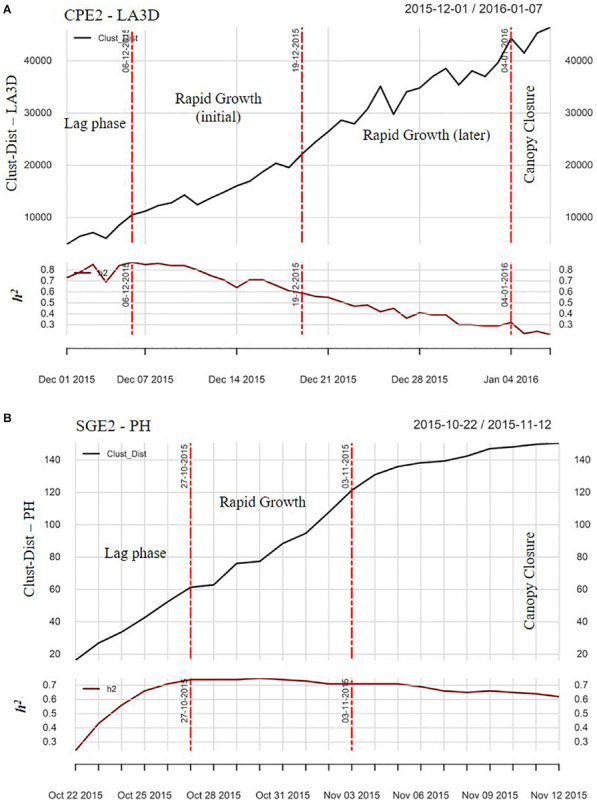
The plots of change point analysis (CPA), illustrating the patterns of daily heritability (*h*^2^) estimate and the Clust-Dist for **(A)** LA3D of CPE2 and **(B)** PH of SGE2. The vertical red lines in the plots denote the “change points” and the annotations between two change points (i.e., within each time-window) denote the corresponding growth phases.

Figure 3 illustrates the TWs obtained after preprocessing and spatial adjustment in a chickpea example (CPE2 LA3D, Figure 3A) and a sorghum example (SGE2 PH, Figure 3B). The TWs essentially represented the initial lag-phase of plant canopy establishment, the phase of rapid canopy expansion and the later vegetative stage during which the canopies closed. Four TWs were obtained in the chickpea example (Figure 3A) and the last one depicted the canopy closure phase during which reliable measurements of the canopy growth parameters were not feasible. Although both the second and third TWs depicted linearly increasing (sometimes erratic) growth, heritability estimates were observed to exhibit a linearly decreasing trend in the third TW. Hence, the second TW was considered optimal for further analysis. The trait development growth curve in the sorghum example (Figure 3B) was both smoother and closer to the sigmoidal pattern compared with the chickpea example (Figure 3A), owing to two factors: (i) better quality raw data in sorghum and (ii) since sorghum plants are larger than chickpea, the former reached a plateau within the time course of the experiment. The second TW in the sorghum example represented the steepest trend in crop growth as well as highest median *h*^2^ estimate, and hence was optimal. The median *h*^2^ (∼0.7) during the OTW of both examples ensured reasonable genotypic variance during that growth phase, which could be suitably leveraged for breeding applications.

### G_*c*_ × TW Analysis

The results of *G*_*c*_ × TW analysis are presented in Tables 7, 8. The effect of TW was found to be highly significant (*p* < 0.001) for all the traits, species, and experiments, while the interaction term had the least significant effect. In terms of crop types, TW explained an average of approximately 85 and 95% of the total sum of squares in chickpea (Table 7) and sorghum (Table 8), respectively. This implied that sorghum exhibits more vigorous transitions in phenotypic development than chickpea. The relative effect of TW was similar for both the traits (LA3D and PH) of sorghum, which exhibited quite close mean sum of square percentage (SS%), i.e., ∼94–95%. However, LA3D (mean SS% of TW ∼87%) in chickpea developed more diversely across growth phases than PH (mean SS% of TW ∼82%). It was further observed that the cluster differences in chickpea were significantly larger than that of sorghum. The relative effect of the *G*_*c*_ × TW interaction was however similar in both the chickpea experiments but differed between the two sorghum experiments. In terms of traits, SS% associated with *G*_*c*_ was again found similar for both LA3D and PH, in sorghum, while it differed marginally between PH and LA3D of chickpea. The average SS% explained by the *G*_*c*_ × TW interaction in chickpea (4%) and sorghum (0.5%) also suggested that chickpea clusters varied more between the TWs than that of sorghum. Hence, it showed that sorghum clusters were probably more consistent over time than the chickpea clusters. These *G*_*c*_ × TW findings thus, corroborated (i) the stability of clustering and (ii) the importance of OTW identification.

**TABLE 7:** The degrees of freedom (Df), sum of squares (SS), percent sum of squares (SS%), mean squares (MS), and the *F*-value (*F-*val) are shown for each source of variation, obtained from the *G*_*c*_ × TW analysis of LA3D and PH of chickpea experiments (CPE1, CPE2).

**Sources of variation**	**CPE1-LA3D**					**CPE1-PH**				
	**Df**	**SS**	**SS%**	**MS**	***F-*val**	**Df**	**SS**	**SS%**	**MS**	***F-*val**
Genotypic clusters (*G*_*c*_)	2	8.06E + 09	7.618	4.03E + 09	1046.981***	2	5.12E + 03	6.907	2.56E + 03	898.522***
Time window (TW)	2	9.24E + 10	87.361	4.62E + 10	12006.383***	2	6.23E + 04	84.098	3.12E + 04	10939.513***
*G*_*c*_ × TW	4	2.02E + 09	1.91	5.05E + 08	131.258***	4	4.23E + 03	5.708	1.06E + 03	371.255***
Residuals	855	3.29E + 09	3.111	3.85E + 06		855	2.44E + 03	3.286	2.85E + 00	
Total		1.06E + 11					74,123			

**Sources of variation**	**CPE2-LA3D**					**CPE2-PH**				
	**Df**	**SS**	**SS%**	**MS**	***F-*val**	**Df**	**SS**	**SS%**	**MS**	***F-*val**

Genotypic clusters (*G*_*c*_)	2	7.48E + 08	4.196	3.74E + 08	603.549***	2	7.20E + 03	10.186	3.60E + 03	1,002.001***
Time window (TW)	2	1.56E + 10	87.476	7.80E + 09	12,583.018***	2	5.71E + 04	80.867	2.86E + 04	7,955.314***
*G*_*c*_ × TW	4	9.55E + 08	5.357	2.39E + 08	385.258***	4	3.25E + 03	4.602	8.13E + 02	226.348***
Residuals	855	5.30E + 08	2.972	6.20E + 05		855	3.07E + 03	4.346	3.59E + 00	
Total		1.78E + 10					70,669			

**p < 0.01.

**TABLE 8:** The degrees of freedom (Df), sum of squares (SS), percent sum of squares (SS%), mean squares (MS), and the *F*-value (*F-*val) are shown for each source of variation, obtained from the *G*_*c*_ × TW analysis of LA3D and PH of sorghum experiments (SGE1, SGE2).

**Sources of variation**	**SGE1-LA3D**					**SGE1-PH**				
	**Df**	**SS**	**SS%**	**MS**	***F-*val**	**Df**	**SS**	**SS%**	**MS**	***F-*val**
Genotypic clusters (*G*_*c*_)	2	3.99E + 08	0.051	1.99E + 08	10.785***	2	3.21E + 04	0.371	1.61E + 04	58.197***
Time window (TW)	2	7.64E + 11	97.215	3.82E + 11	20,665.584***	2	8.31E + 06	95.947	4.16E + 06	15,063.085***
*G*_*c*_ × TW	4	3.55E + 08	0.045	8.87E + 07	4.802**	4	3.67E + 03	0.042	9.18E + 02	3.325*
Residuals	1,143	2.11E + 10	2.689	1.85E + 07		1143	3.15E + 05	3.64	2.76E + 02	
Total		7.86E + 11					8,665,647			

**Sources of variation**	**SGE2-LA3D**					**SGE2-PH**				
	**Df**	**SS**	**SS%**	**MS**	***F*-val**	**Df**	**SS**	**SS%**	**MS**	***F*-val**

Genotypic clusters (*G*_*c*_)	2	2.86E + 09	0.911	1.43E + 09	138.322***	2	3.38E + 04	1.153	1.69E + 04	186.857***
Time window (TW)	2	2.98E + 11	94.953	1.49E + 11	14,413.423***	2	2.74E + 06	93.53	1.37E + 06	15,154.881***
*G*_*c*_ × TW	4	1.17E + 09	0.372	2.92E + 08	28.255***	4	5.25E + 04	1.789	1.31E + 04	144.959***
Residuals	1,143	1.18E + 10	3.764	1.03E + 07		1,143	1.03E + 05	3.527	9.05E + 01	
Total		3.14E + 11					2,932,587			

*p < 0.05; **p < 0.01; ***p < 0.001.

## Discussion

### Effect of Data Preprocessing

As observed from the simulation results (Table 5), the use of a strategy combining outlier detection and missing value imputation (S8) performed the best in most of the cases. Especially in the datasets affected by both missing values and contaminated data, which increased the number of outliers. Such outcomes were also reflected in the real data analyses, but to a lesser extent. For example, the positive effect of outlier detection was noticed only in the CP PH between experiment comparison (Table 4). We could also detect a positive effect of missing value imputation on *h*^2^ in the CV performed on dataset CPE1 PH (Table 3). Those results were all observed in the chickpea data that contained more missing and extreme values. Therefore, data preprocessing we propose is mostly useful for data characterized by a large amount of noise, which can be characteristics of data generated on outdoors HTP platforms.

The difference between the simulations results and the real data evaluation can be explained by two factors. The first one is the amount of contaminated data needed to observe significant differences. As per the simulations, we could start to observe significant differences between the procedures when the proportion of contaminated data was at least 10–20%. Whereas, in the real data, the maximum proportion of the values that could be considered outliers was only 3.6%, which certainly represented lower levels of data contamination. Another reason is that in the CV process, the days were randomly selected. While some of those days could have many contaminated values, others might have had a few, and averaging of those results could cancel out the effects.

It was further noticed from the simulations that, the sole presence of missing values is not a big problem. All the methods were able to obtain particularly good results even in scenarios involving up to 50% of missing values. In those scenarios, we could see that strategy using only imputation (S7) was very performant. Such a result supports the idea that PMM is an appropriate method for imputation in HTP time series data. It is similar to previous findings showing the robustness of PMM against large rate of missing values (Marshall et al., 2010; Kleinke, 2018).

The presence of extreme values in the data is more problematic than the one of missing values. According to the simulations, the procedures could still produce reasonably good results up to 30% of contaminated data if there are no missing values. The presence of missing values along with noise results in reduced performance of the procedures, which becomes critical beyond 20–30% of contaminated and or missing values. Although these results are quite informative, the simulations presented in this article are derived from a single real-data situation. The users could apply the same procedure on their data to determine the limitations specific to their situation. Here, we also want to highlight the fact that, in this study, we globally did not experience a situation where the leaf area was substantially dropping over time. However, as we could observe in Figure 2 (SG), the leaf area tended to decrease on the last day. This could be the sign that the plant started to reach the plateau phase. We could also imagine that in other conditions like water stress, the total leaf area would decrease at a certain point due to leaf senescence. In that case, we would use another model to fit the data. For example, we could use a segmented linear function with one linear function describing the growth phase and another function describing the decrease phase when leaf senescence dominates the process. However, currently those analyses are beyond the scope of this work.

The simulation results also supported the use of S8 combining outlier detection and missing value imputation in separate steps over S9, which performs imputation during the estimation process and detects outliers based on the mixed model residuals. In all scenarios where noise was added to the data, S8 obtained better results than S9. We could also observe such a result but with a reduced difference in the between experiment comparison (CP PH) and the goodness of fit results of the logistic curve (e.g., CPE1 PH).

From a methodological point of view, the difference between S9 and S8 is due to the fact that S9 uses data only from a single day, while S8 benefits from the information present in the whole time series to impute the missing values. In S8, the possibility to use past and present information allows to build highly accurate prediction for missing values using the growth trend information. In S9, however, missing value imputation is done using the spatial and genetic information of a single day, which makes it more sensitive to the presence of extreme values on that day. The sequential application of outlier detection and imputation by considering the similar values in the phenotypic time series is therefore, a simple yet effective strategy to restore information in contaminated data.

### Effect of Spatial Adjustment

The use of spatial adjustment had a positive and significant effect in almost all data analysis evaluations. Such a result can be explained by the fact that data measured in outdoor or field conditions like on the LeasyScan platform are subject to a substantial amount of spatial variation. More precisely, we could see in the SpATS results (Supplementary Figure 3) that the replicates located closer to the wall (e.g., Supplementary Figure 3C) exhibited faster phenotypic development (represented in yellow), likely because of overnight dissipation of the heat accumulated in the concrete wall, compared with the ones located in the middle or on the opposite edges of the platform. Furthermore, the complexity of such spatial interaction was found to increase as the crops grew larger (Supplementary Figure 4). As far as we know, the use of spatial adjustment to process HTP data from an outdoor platform has not been evaluated in previous studies. Extending the results from Velazco et al. (2017), we have shown that the application of the SpATS model in a routine analysis is of great value to account for spatial variation and increase the genetic heritability. The high goodness of fit values for the description of the G-BLUEs time series using a logistic curve (Table 6) illustrates the overall quality of the proposed procedure and its ability to obtain biologically relevant profiles of plant growth traits.

### Temporal Analysis of G-BLUEs

Phenotyping of complex traits, e.g., those associated with canopy development, in a diverse set of genotypes is often found to be challenging under non-controlled environments, due to the differences in the responses of genotypes to ambient conditions. This eventually results in reduced *h*^2^ of the growth-related traits (Rebetzke et al., 2014). van der Heijden et al. (2012) have discussed the need to exploit temporal information for improving phenotyping efficiency of traits like total leaf area. A few studies have considered temporal plant phenotyping for assessing genotypes based on temporal patterns of phenotypic development (van Dusschoten et al., 2016; Das Choudhury et al., 2017). However, to the extent of our knowledge, a systematic approach to determining an OTW where *h*^2^ and genetic diversity are maximized, has not yet been proposed. The incorporation of CPA in this study is therefore an effort to enable an automated and systematic extraction of temporal information from phenotypic data, such that both genetic diversity as well as trait heritability are maximized. Hence, obtaining smoother G-BLUEs time series was also beneficial for identifying the OTW, since an important aspect of the pipeline was to differentiate temporal sections of the G-BLUEs time series with maximum genotypic resolution.

From Figure 3A, phenotypic data toward the later portions of the chickpea experiment were erratic due to overlap of crop canopies, especially during the rapid growth phase, resulting in consequent loss of sensor resolution (Vadez et al., 2015; Zhokhavets et al., 2018) and lowered *h*^2^. In the chickpea example (Figure 3A), although both 2nd and 3rd TWs biologically represented the rapid growth phase, a declining trend of *h*^2^ was found in the 3rd TW. Therefore, the 2nd TW with the highest genotypic resolution was chosen as optimal. The selected OTW confirmed with our initial hypothesis, and as suggested by van der Heijden et al. (2012) that—the most suitable time to phenotype plants is during initial period of rapid development, when plants are less high and dense, i.e., before the canopy closure phase. Phenotypic data within the OTW of each trait were further utilized to cluster genotypes based on the similarity in their canopy growth or vigor patterns.

The stability of the clusters across the TWs (Tables 7, 8) was also shown for each of the canopy-growth traits, since the interaction effect was found to be small compared with the TW and cluster effect. Minimal interaction effect achieved through OTW cluster-based *G*_*c*_ × TW analysis also implies more predictable performance of the trait of interest for each genotype within a particular cluster (Sorrells, 2015). Thus, by fitting the two-way ANOVA model, we could verify the biological assumption that—most of the phenotypic variation comes from the difference in growth stage (TW), to a reduced but significant extent from the genotypic difference (*G*_*c*_) and to a lesser extent from difference in genotype modified by the time (*G*_*c*_ × TW). However, several studies have reported that large interaction effects that tend to complicate results interpretation, since phenotypic expression (and hence, ranking) of genotypes would differ across environments (Kaya et al., 2006; Tulu and Wondimu, 2019). Those studies primarily consider genotypic analysis across environments, and not growth-phases, which could also potentially alter the ranking of genotypes. Therefore, clustering similar genotypes based on the results of CPA was performed, since information contained in temporal datasets is generally non-homogeneous throughout the crop growth stage due to changing patterns of phenotypic development (Shi et al., 2016).

Obtaining such consistent results from the temporal analyses could be ascribed to the effects of systematic data treatment. Those procedures helped produce “clean” time series that were more representative of the underlying biological mechanism, in terms of both trait growth and the differences among genotypes. While the preprocessing procedures helped improve the possibility to detect some continuity in the time series (important to detect the effect of time), spatial adjustment helped remove variability due to spatial heterogeneity and then estimate the genotypic effect. Consequently, the improved estimate allowed detecting the ‘true’ expected differences between genotypes. Therefore, we believe that with the proposed procedure the resultant data will be representative of their expected behavior in future experiments, which is essential for selection.

## Conclusion

Many HTP platforms have been established to perform large-scale experiments, where hundreds of genotypes are screened simultaneously. Although such platforms can generate large datasets, their usage is frequently limited because of the non-availability of convenient and suitable HTP data analysis pipelines. Hence, the sequential analytical pipeline (SpaTemHTP) presented here addresses these limitations and was developed to efficiently process data generated at outdoors platforms, that are characterized by an important amount of exogenous variation. The pipeline embeds a modular design to process raw HTP data for calculating spatially adjusted genotypic estimates, which is generally a complex procedure requiring knowledge about handling of spatial variability using mixed models. The significance of spatial adjustment in enhancing the quality of model estimates was also demonstrated through extensive testing of the pipeline for different species, traits, and experiments. Using a sequence of steps including outlier detection, missing value imputation, and spatial adjustment (S8) allowed to obtain smoother G-BLUEs time series that conformed to the biological expectation, better than the ones produced by a single-step mixed-model approach (S9). Additionally, S8 is conceptually simpler than S9 and computationally less intensive. The usefulness of our approach was particularly relevant for raw data with a large amount of noise, since it could be shown that the pipeline can easily handle up to 50% of missing values with minor impact on the results. We could also show that our procedure produces acceptable results up to 20–30% of contaminated data. Furthermore, the modular design of the pipeline allows to choose the set of operations as per user requirements, e.g., some dataset might not require preprocessing or CPA.

The genotype adjusted means data produced by the pipeline allows the user (breeder) to get quality data for immediate analysis (genotype comparison) or for further analysis like statistical genetics models (QTL, GWAS, or genomic prediction models). Hence, this automated procedure would also be extremely useful for larger data workflow processing strategy. Through CPA, we also showed the use of adjusted means to identify important time sections during an experiment, which provides a basis for further dissection of the genetic components in terms of *G*_*c*_ × TW. For example, the G-BLUEs obtained at different OTW could be used in a multitrait QTL analysis that would help to model genetic effects that take into consideration the longitudinal nature of trait development. This genotype by time analysis could also be further extended to a genotype by time by environment analysis that will help to understand the environmental effect on trait development. Thus, it can be concluded that the proposed pipeline can be employed particularly for large-scale outdoors HTP data and be beneficial for several biological applications.

## Data Availability Statement

The raw data supporting the conclusions of this article will be made available by the authors, without undue reservation, to any qualified researcher.

## Author Contributions

SK: conceptualization, methodology development, coding R scripts, validation, writing original draft, and review and editing. VG: validation, coding, and review and editing of the draft. JK and VV: conceptualization, methodology development, supervision, and review and editing of the draft. SD: supervision, and review and editing of the draft. RT: conceptualization, validation, coding, and review and editing of the draft. HI: conceptualization, validation, and review and editing of the draft. MU: experimental data. JA: conceptualization, supervision, and review and editing of the draft. All authors contributed to the article and approved the submitted version.

## Conflict of Interest

The authors declare that the research was conducted in the absence of any commercial or financial relationships that could be construed as a potential conflict of interest.

## Footnotes

1 www.gems.icrisat.org/LeasyScan
