# Supplementary material for: SpaTemHTP: A Data Analysis Pipeline for Efficient Processing and Utilization of Temporal High-Throughput Phenotyping Data
Source: Front Plant Sci. 2020 Nov 20;11:552509. doi: 10.3389/fpls.2020.552509 (PMC7714717; doi:10.3389/fpls.2020.552509)
Supplement: Supplementary file 1 [file Data_Sheet_1.PDF]

Supplementary Material

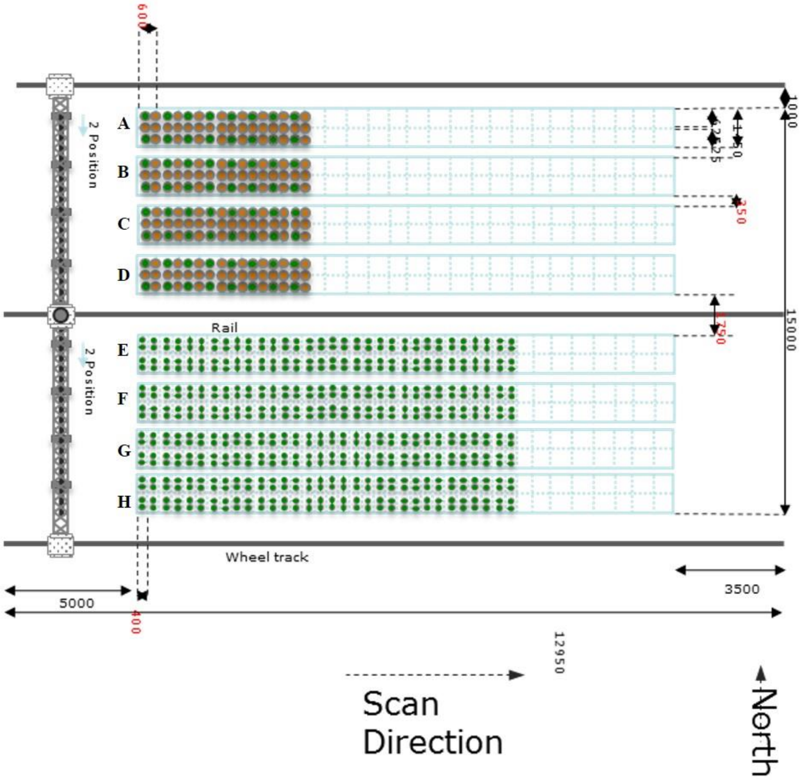

Supplementary Figure 1. Schematic representation of the LeasyScan (LS) HTP platform. The scanning direction is from West to East, and the concrete walls run along the edges of the platform. LeasyScan has 8 columns (also called trenches, denoted as A (1,2) to H (1,2), in the North to South direction), and each comprises 288 sectors (the micro-plots). There are accordingly 8 PlantEye® 3D laser scanners atop each column, traversing from West to East, which measure the crop parameters (LA3D, PLA and PH). There are 12 sensors (to record ambient T and RH%) placed at different locations in each of the columns.

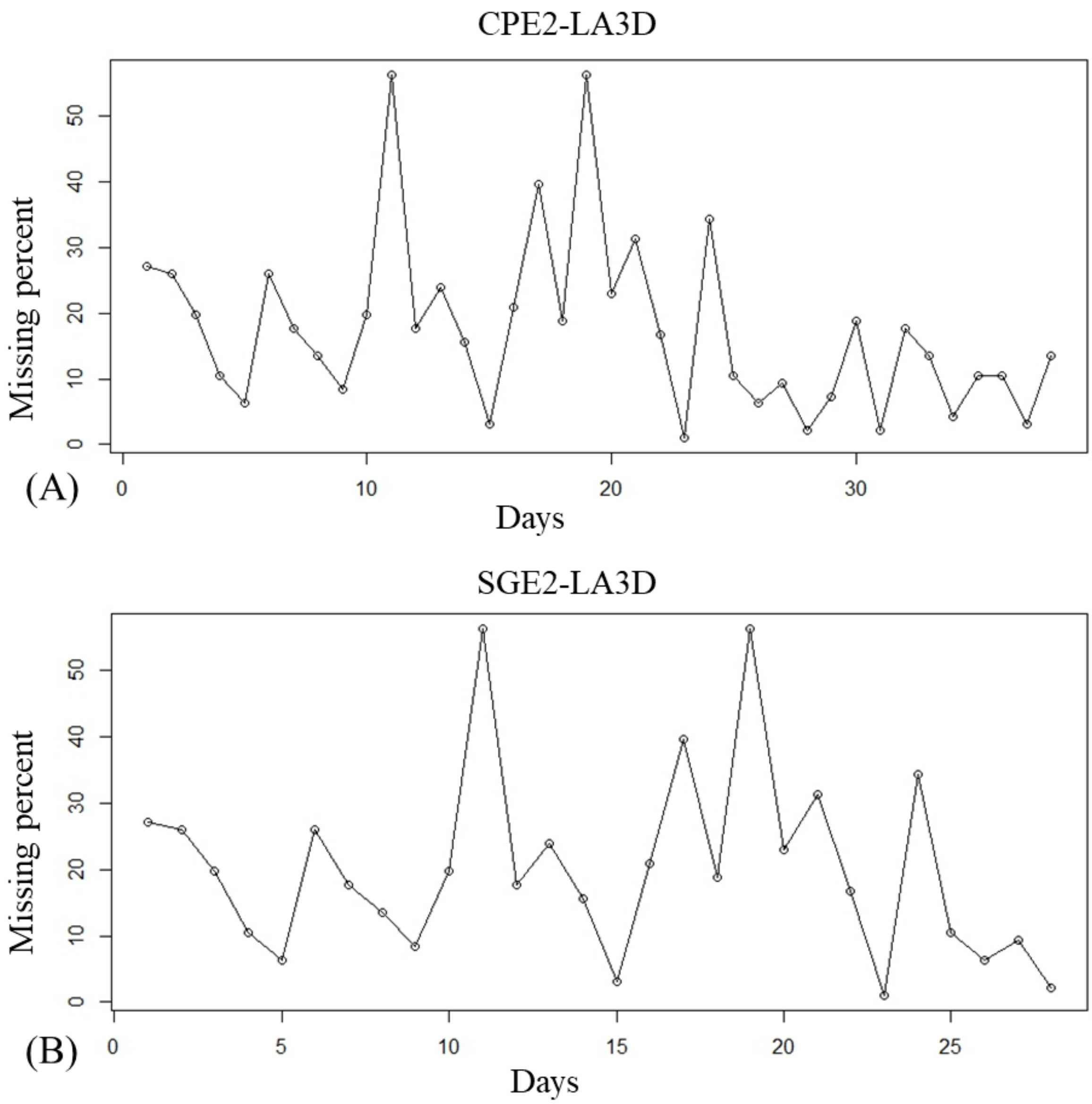

Supplementary Figure 2. Total number of missing observations in the raw data, plotted across each experiment, CPE2 (A) and SGE2 (B) for the trait LA3D.

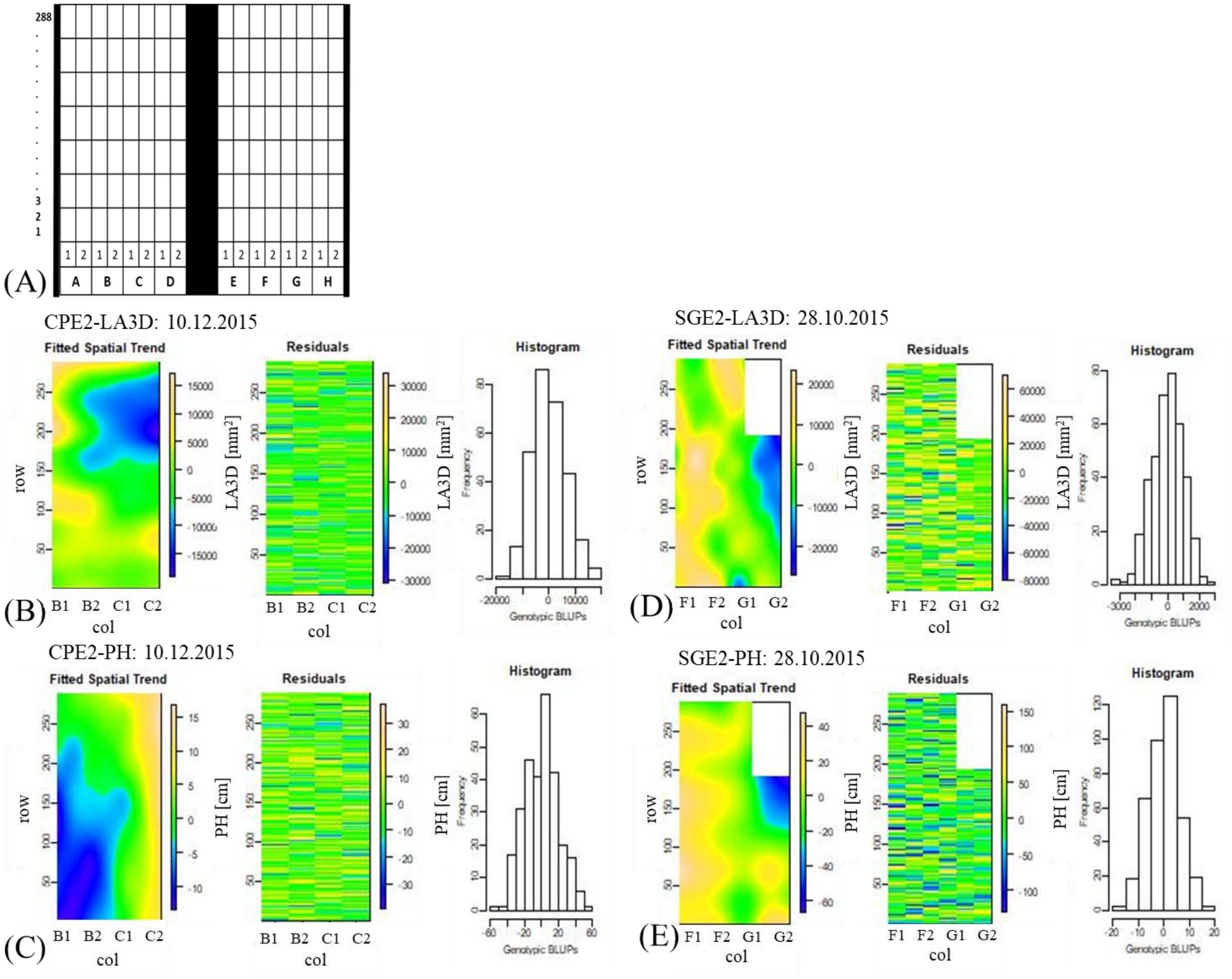

Supplementary Figure 3. Schematic diagram of the platform is shown in (A), the letters (A, B, …, H) within it denote the trenches, and the thick lines along the edges and the center represent the concrete walls. The plots in (B) and (C) denote a spatially fitted trend, the residuals, and the histogram of genotypic BLUPs obtained from the SpATS model for LA3D [mm^2] and PH [cm] values of chickpea (CPE2) for 10.12.2014. Similarly, plots (d) and (e) denote the same for sorghum (SGE2) for 28.10.2015. The replicates located closer to the wall exhibited faster phenotypic development (yellow regions), compared to the ones located in the middle or on the opposite edge of the platform (blue regions). Sorghum PH with a more irregular spatial-surface than that LA3D denoted a greater spatial interaction complexity. In chickpea, complexity of spatial interaction was more in LA3D than PH.

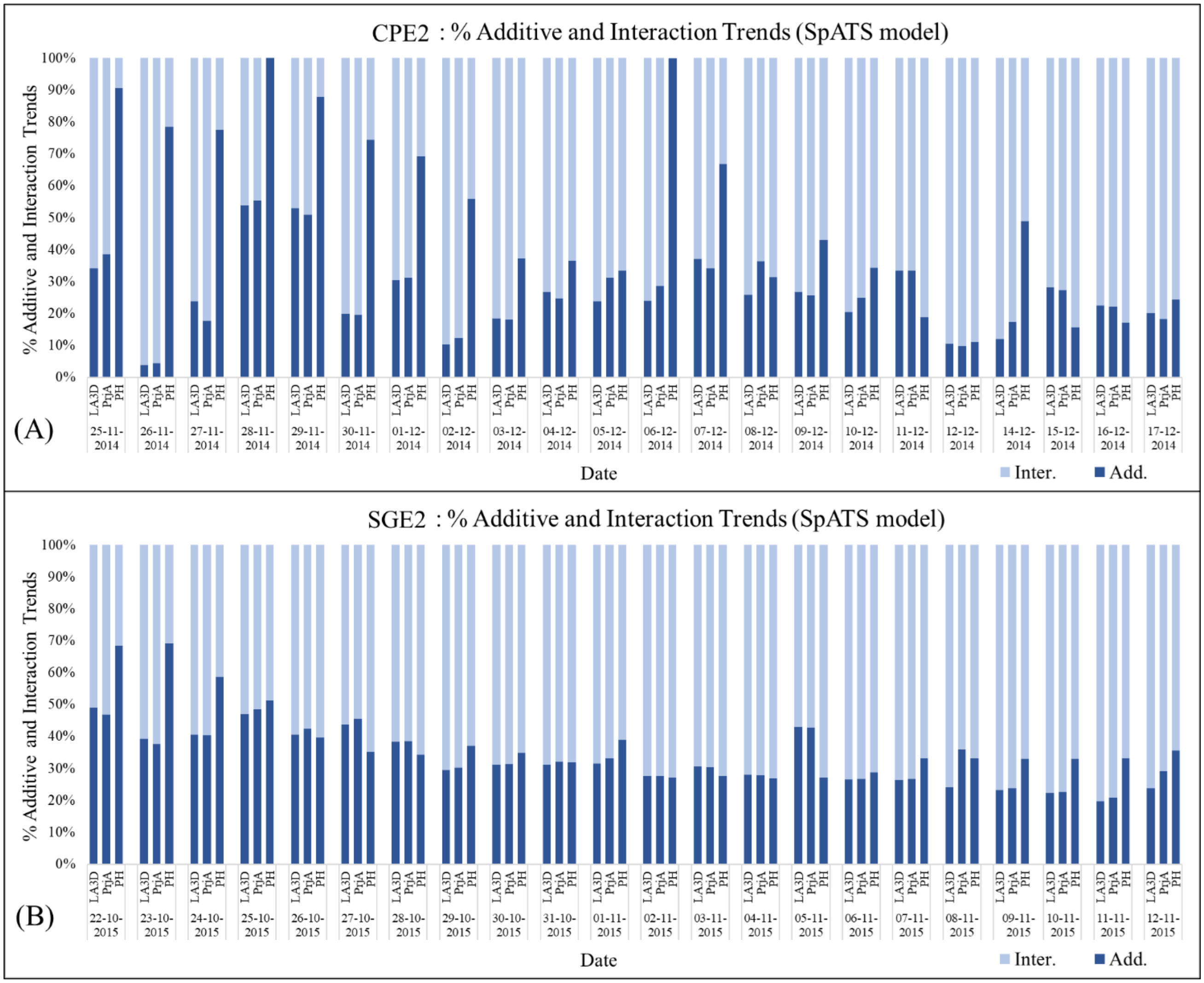

Supplementary Figure 4. Stacked bar plot representation of the differences in the proportions of additive (Add.) and interactive (Inter.) spatial trends, obtained from the SpATS model for the traits – LA3D, PLA and PH, of (A) chickpea (CPE2) and (B) sorghum (SGE2), that indicated an increase in interaction complexity towards the later part of the vegetative stage in both species.
